# Comparison of MRI-, CT- and PET-based anatomical standardization for Centiloid scale calculation in [^18^F]florbetapir positron emission tomography

**DOI:** 10.1007/s12149-025-02134-4

**Published:** 2025-12-04

**Authors:** Hirofumi Yamada, Kota Yokoyama, Jun Oyama, Junichi Tsuchiya, Yoichiro Nishida, Nobuo Sanjyo, Masahito Yamada, Takanori Yokota, Ukihide Tateishi

**Affiliations:** 1 Department of Diagnostic Radiology, Institute of Science Tokyo, Bunkyo-ku, Tokyo, Japan; 2 Department of Neurology and Neurological Science, Institute of Science Tokyo, Bunkyo-ku, Tokyo, Japan; 3 Division of Neurology, Department of Internal Medicine, Kudanzaka Hospital, Chiyoda-ku, Tokyo, Japan

**Keywords:** Amyloid PET, Centiloid scale, Anatomical standardization, Florbetapir

## Abstract

**Objective:**

The Centiloid (CL) method is a standardized quantitative approach for amyloid positron emission tomography (PET) that involves the anatomical standardization of PET images. The Global Alzheimer’s Association Interactive Network recommends performing this anatomical standardization using contemporaneously acquired three-dimensional (3D) T1-weighted magnetic resonance imaging (MRI). However, in clinical practice, contemporaneous 3D T1WI is not always available due to outdated imaging or missing sequences. Recently, alternative methods utilizing low-dose computed tomography (CT) scan images from PET/CT scan or PET images themselves for anatomical standardization have been proposed and implemented in commercially available software. This study aimed to compare the CL obtained via MRI-based standardization with CT scan-based and PET-only standardizations. Further, the clinical applicability of these alternative methods was assessed.

**Methods:**

We retrospectively analyzed 68 patients who underwent [18 F]florbetapir PET/CT scan imaging. The CL were calculated using two commercially available software programs—AMYclz^®^ Neuro (PDR Pharma, Tokyo) and BRAINEER^®^ Model A (Splink, Tokyo)—under three conditions: anatomical standardization with MRI, CT scan, and a PET-derived template. The correlations and differences among these methods were evaluated.

**Results:**

68 patients (age: 70.8 ± 11.2) were included in the analysis. The median CL was 33.87 (3.53–76.57). CT scan-based standardization had lower CL than MRI-based standardization (mean difference: −5.9 ± 4.8). Meanwhile, PET-only standardization had slightly lower CL than MRI-based standardization (-2.1 ± 11.0). The differences in the CL were generally within ± 15. In cases with evidently positive (CL ≥ 50) or clearly negative (CL ≤ 5) findings, the omission of MRI did not affect exceeding the threshold.

**Conclusions:**

When contemporaneous 3D T1WI is not available, CT scan-based or PET-only anatomical standardization can be a practical alternative for the qualitative assessment of amyloid PET. However, users should recognize that CT scan-based methods have a systematic tendency to underestimate CL values compared with MRI-based methods. Meanwhile, PET-only methods have a tendency to slightly underestimate CL values. The omission of MRI may be acceptable for clear positive or negative cases. However, caution is required for borderline cases, particularly in situations requiring precise quantification such as treatment monitoring.

**Supplementary Information:**

The online version contains supplementary material available at 10.1007/s12149-025-02134-4.

## Introduction

Alzheimer’s disease (AD) is characterized by a long preclinical phase during which amyloid-beta accumulation occurs in the brain, often years before symptom onset [1]. Amyloid positron emission tomography (PET) enables the sensitive detection of this early pathological change [2]. Traditionally, amyloid PET interpretation was dependent on visual assessment or semiquantitative methods such as the standardized uptake value ratio (SUVR) [3]. More recently, the Centiloid (CL) scale has been developed and widely adopted to facilitate standardized comparisons across different tracers and imaging systems [4]. The CL is continuous, with the average uptake set at 0 in young adults who are amyloid-negative and at 100 in patients with typical AD. It is now used not only for qualitative classification (positive/negative) but also for the prognostic assessment and evaluation of treatment response, thereby underscoring the need for an accurate and reproducible CL quantification [5].

According to the Global Alzheimer’s Association Interactive Network (GAAIN), anatomical standardization using contemporaneously acquired three-dimensional (3D) T1-weighted magnetic resonance imaging (MRI) is recommended for CL calculation [4]. Nonetheless, in clinical practice, performing such type of MRI at the time of PET imaging is often challenging due to scheduling limitations, missing data, or the absence of appropriate sequences. In such cases, the standard CL processing pipeline cannot be applied. MRI-based registration remains the reference standard. Nevertheless, low-dose computed tomography (CT) scan images acquired during PET/CT have been utilized as alternative anatomical references for spatial normalization [6–9]. A previous study using [18 F]flutemetamol has shown that CT scan-based CL calculation is feasible. However, it may slightly underestimate CL values compared with MRI-based methods [8].

In addition to CT scan-based approaches, recent developments have enabled CL calculation without any anatomical reference, using PET-only registration to tracer-specific templates [10–13]. Imabayashi et al. have reported that the CL calculated from [^18^F]flutemetamol PET images without morphological images had a remarkably high concordance with standardized uptake value ratios (SUVR) derived from morphological registration [12].

Further, Cong Shang et al. have revealed a high degree of consistency in CL values across three different commercial software tools [14].

In light of these findings, it is assumed that anatomical standardization may not always be essential when using commercial software. Such approaches have been incorporated into commercially available software packages, thereby facilitating practical application in settings where MRI is not available. This study investigated the clinical utility of two software tools—AMYclz^®^ Neuro (PDR Pharma, Tokyo, Japan; available at https://www.pdradiopharma.com/) [15] and BRAINEER^®^ Model A (Splink, Tokyo, Japan; available at https://www.splinkns.com/information/)—for CL quantification using [^18^F]florbetapir PET. In particular, CL values obtained via MRI-based, CT scan-based, and PET-only pipelines were compared to evaluate the feasibility of omitting anatomical standardization under specific clinical conditions.

## Materials and methods

### Research participants

This was a retrospective cohort study involving patients who underwent amyloid PET/CT scan using [^18^F]florbetapir between January 2023 and August 2024. Cases prior to January 2023 were excluded from the study because they were imaged using non-semiconductor PET/CT systems. The institutional ethical committee approved the current study (approval no. I2024-048) in accordance with the Good Clinical Practice guidelines and the 1964 Declaration of Helsinki. As this was a retrospective study, informed consent was obtained using an opt-out method.

### Imaging protocol

[^18^F]Florbetapir was injected intravenously as a slow bolus into an antecubital vein at a mean ± standard deviation (SD) dose of 370 ± 10 MBq (range: 350–390). PET imaging was initiated 40 min after injection and was continually administered for 20 min. All PET scans were performed using a semiconductor PET/CT scanner (Cartesion Prime; Canon Medical Systems, Japan). Image reconstruction was conducted using the three-dimensional ordered subset expectation maximization (3D-OSEM) algorithm with the following parameters: image matrix size, 128; field of view, 256 mm; 12 subsets; 6 iterations; and a 4-mm full width at half maximum Gaussian post-filter. A low-dose CT scan was obtained (120 keV, 100 mA, and 1.0 s rotation) for attenuation correction. The resulting voxel size was 2.0 × 2.0 × 3.0 mm.

### CL scale calculation

The CL was calculated using two different commercially available software: AMYclz^®^ Neuro (PDR Pharma, Tokyo, Japan; available at https://www.pdradiopharma.com/) [15] and BRAINEER^®^ Model A (Splink, Tokyo, Japan; available at https://www.splinkns.com/information/). AMYclz^®^ Neuro utilized both PET and CT scan/MRI data. Meanwhile, BRAINEER^®^ Model A employed either PET-only or PET with MRI.

For the AMYclz^®^ Neuro pipelines (AMYclz-CT and AMYclz-MRI), the anatomical normalization was performed to the Montreal Neurological Institute (MNI) standard space using the unified segmentation algorithm implemented in SPM12 (Wellcome Department of Imaging Neuroscience, London, UK). Anatomical standardization proceeds through the following steps: first PET and MRI (or CT) images are reoriented and coregistered; second MRI (or CT) images are nonlinearly warped into MNI space using SPM12 unified segmentation, which combines tissue classification and spatial normalization; last the deformation fields derived from MRI (or CT) normalization are applied to the PET images, resulting in anatomically standardized PET data.

BRAINEER^®^ Model A with MRI Pipeline (BRAINEER-MRI) performed anatomical normalization to the MNI 152 T1WI atlas using the unified segmentation algorithm implemented in SPM12. Anatomical normalization proceeded as follows: MRI images were aligned to the standard brain (MNI 152) using rigid transformation with SPM coregister to match the AC-PC line; Next, the PET image is aligned to the MRI image using SPM coregister via a rigid transformation;　Subsequently, the MRI image is aligned to the standard brain in MNI space using DARTEL; Finally, the DARTEL transformation field is applied to the rigidly transformed PET image to obtain anatomically normalized PET data.

In BRAINEER^®^ Model A with PET Pipeline (BRAINEER-PET), anatomical normalization to multiple PET atlases (templates of average images from multiple subjects) was performed using the unified segmentation algorithm implemented in SPM12. Anatomical normalization proceeds as follows: Align the PET image to the PET standard brain (PET atlas) using rigid transformation via SPM Coregister to match the AC-PC line; then, align the PET image to the PET standard brain in MNI space using the spatial normalize toolbox (affine + discrete cosine transform (DCT) based registration) to obtain anatomically normalized PET data.

The optimization step method employed the Mean Squared Error (MSE) approach (J. Ashburner et al. 1999 [16]), the DARTEL method (J. Ashburner. 2007 [17]), and the Normalized mutual information method (A. Collignon et al. 1995 [18].).

Both the AMYclz Neuro^®^ pipeline and the BRAINEER^®^ Model A pipeline calculate CL values in MNI space from spatially normalized PET images. Based on the GAAIN Centiloid standard VOI, the software applies a global cortical target VOI (CTX VOI) and a whole-cerebellum reference VOI using SPM12. The standardized uptake value ratio (SUVR) was converted to CL units using the following equation (Navitsky et al. [19]): CL = 175.2 × SUVR − 182.2.

No specific MRI models were restricted because clinical MRI images were used. For MRI images, we used the images taken prior to PET imaging and closest to the PET imaging date. For CT images, we used images acquired simultaneously with the PET imaging.

## Visual assessment

By retrospectively reviewing the image interpretation reports, amyloid accumulation was assessed through visual evaluation and classified as either Positive or Negative. The images were reviewed by two physicians who are both radiologists and nuclear medicine specialists, and who specialize in neuroradiology.

### Statistical analysis

Categorical data were presented as the absolute and relative frequencies, and continuous data were expressed as the medians and interquartile ranges (IQR). Scatter plots were generated to assess the concordance between the CL scale calculated using AMYclz^®^ Neuro with CT scan, AMYclz^®^ Neuro with MRI, BRAINEER^®^ Model A with PET-only, and BRAINEER^®^ Model A with MRI. The Bland**–**Altman analysis was performed to evaluate systematic errors. The mean CL values were compared by paired t-tests for each analysis method. The significance level was set to 0.025 to account for multiple comparisons. The minimum sample size required to detect a difference equivalent to one-third of the standard deviation of CL, with an α error of 0.05 and a power of 0.8, was 73 cases. The sample size of 68 cases described subsequently was deemed adequate.

All statistical analyses were performed using EZR (Easy R), a graphical user interface for R developed for medical statistics, based on R Commander (version 1.68; available at https://www.jichi.ac.jp/saitama-sct/SaitamaHP.files/statmedEN.html).

## Result

### Characteristics of the patients

In total, 68 patients underwent amyloid PET/CT scan with [^18^F]florbetapir. The median age of the patients was 74 (IQR: 65–79) years, and 32 were men. The median MMSE was 26 (IQR: 24–28.25.25). The median CL scale was 33.87 (IQR: 3.53–76.57) (calculated with AMYclz^®^ Neuro).

The pre-examination clinical diagnoses were as follows: 16 cases of Alzheimer’s disease and suspected Alzheimer’s disease, 1 case of Alzheimer’s disease with amyloid angiopathy, 1 case of Alzheimer’s disease with normal pressure hydrocephalus, 35 cases of mild cognitive impairment (MCI), 4 cases of preclinical AD, and 8 cases with no diagnosis recorded.

A total of 49 MRI examinations were included in this study. Among these, 6 scans were acquired using a 1.5-Tesla system and 43 scans using a 3-Tesla system. Detailed imaging parameters ware summarized in Supplementary Table 1. The median interval between MRI and PET imaging was 138 days (interquartile range, 60–265.5 days). The CT and PET scans were performed on the same day.

### CL scale calculations and comparisons

**Fig. 1:**
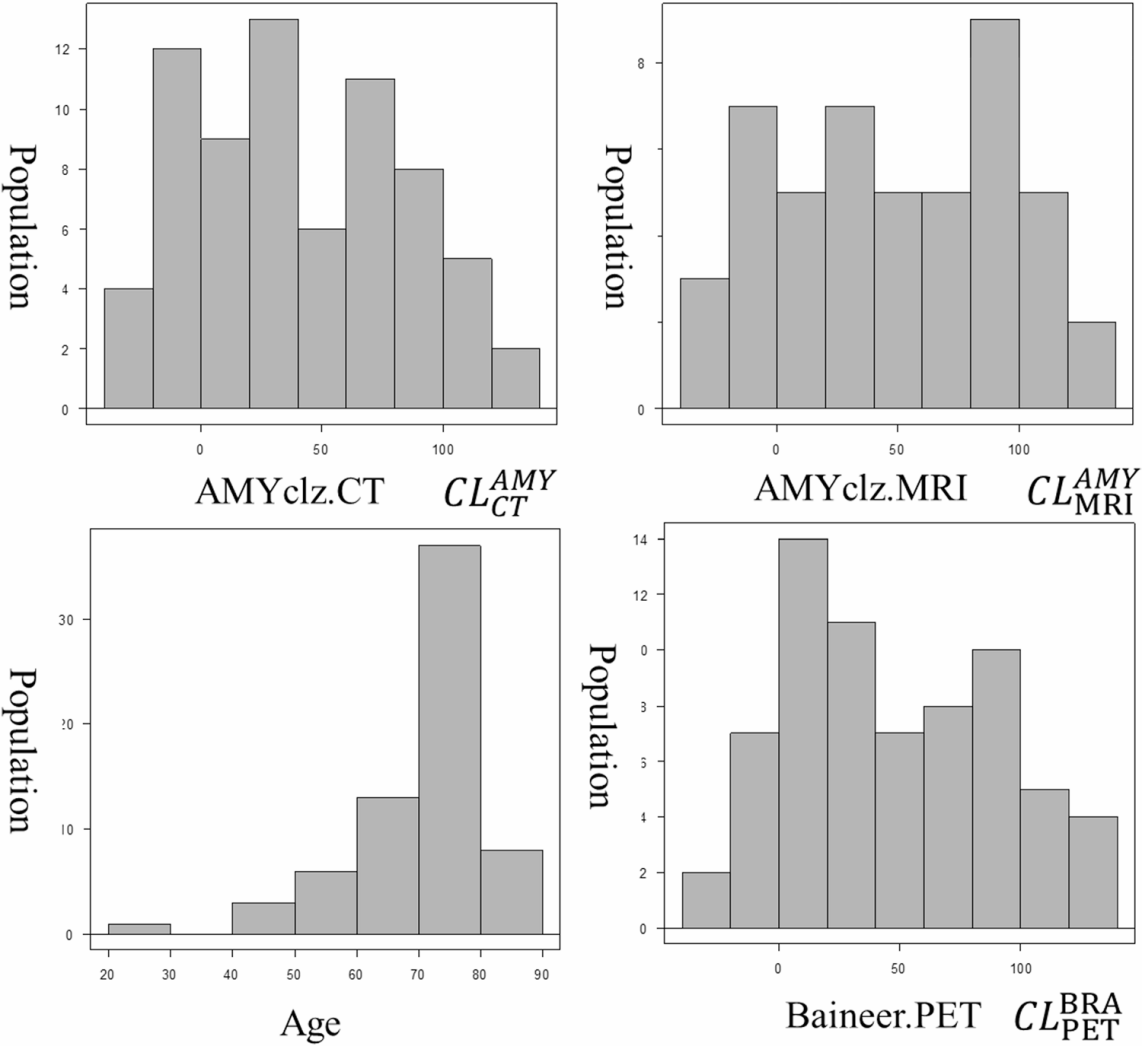
Distribution of age and their Centiloid scale with the patients

The CL scale was calculated using AMYclz^®^ Neuro with CT scan, AMYclz^®^ Neuro with MRI, BRAINEER^®^ Model A with PET-only, and BRAINEER^®^ Model A with MRI (Fig. 1). The median CL scale values were as follows:

- AMYclz^®^ Neuro with CT scan: 33.87 (IQR: 3.53–76.57).
- AMYclz^®^ Neuro with MRI: 43.44 (IQR: 8.88–85.66).
- BRAINEER^®^ Model A with PET-only: 39.76 (IQR: 11.94–81.73).
- BRAINEER^®^ Model A with MRI: 56.51 (IQR: 9.67–90.89).

### Correlation and Bland–Altman analyses

Strong correlations were observed between the different methods.

**Fig. 2:**
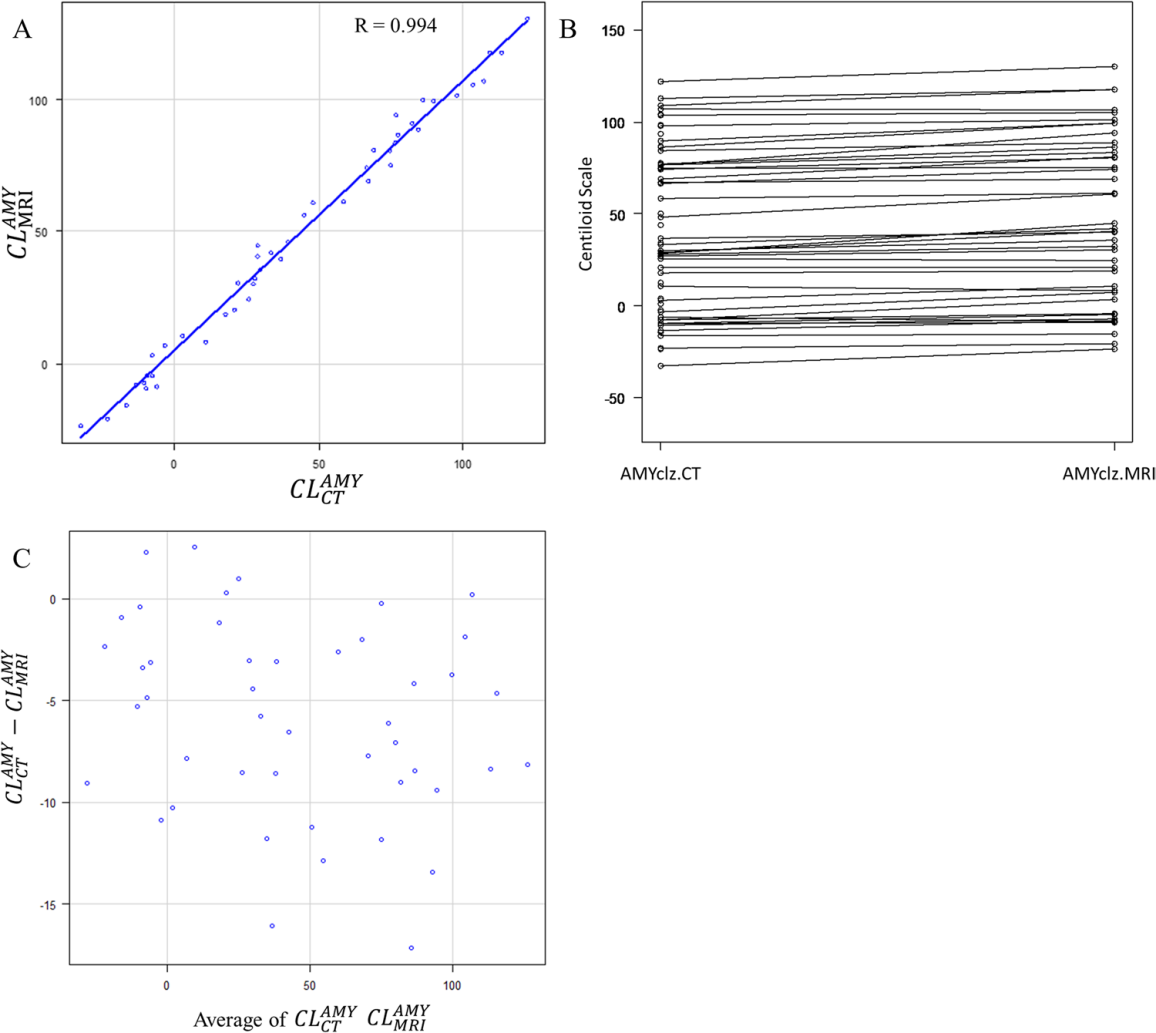
Comparison of AMYclz^®^ Neuro analysis with CT scan and AMYclz^®^ Neuro analysis with MRI. A strong correlation was observed, with a correlation coefficient of 0.994 (**A**) there was a tendency toward underestimation of the Centiloid scale in CT scan cases (**B** and **C**)

**Fig. 3:**
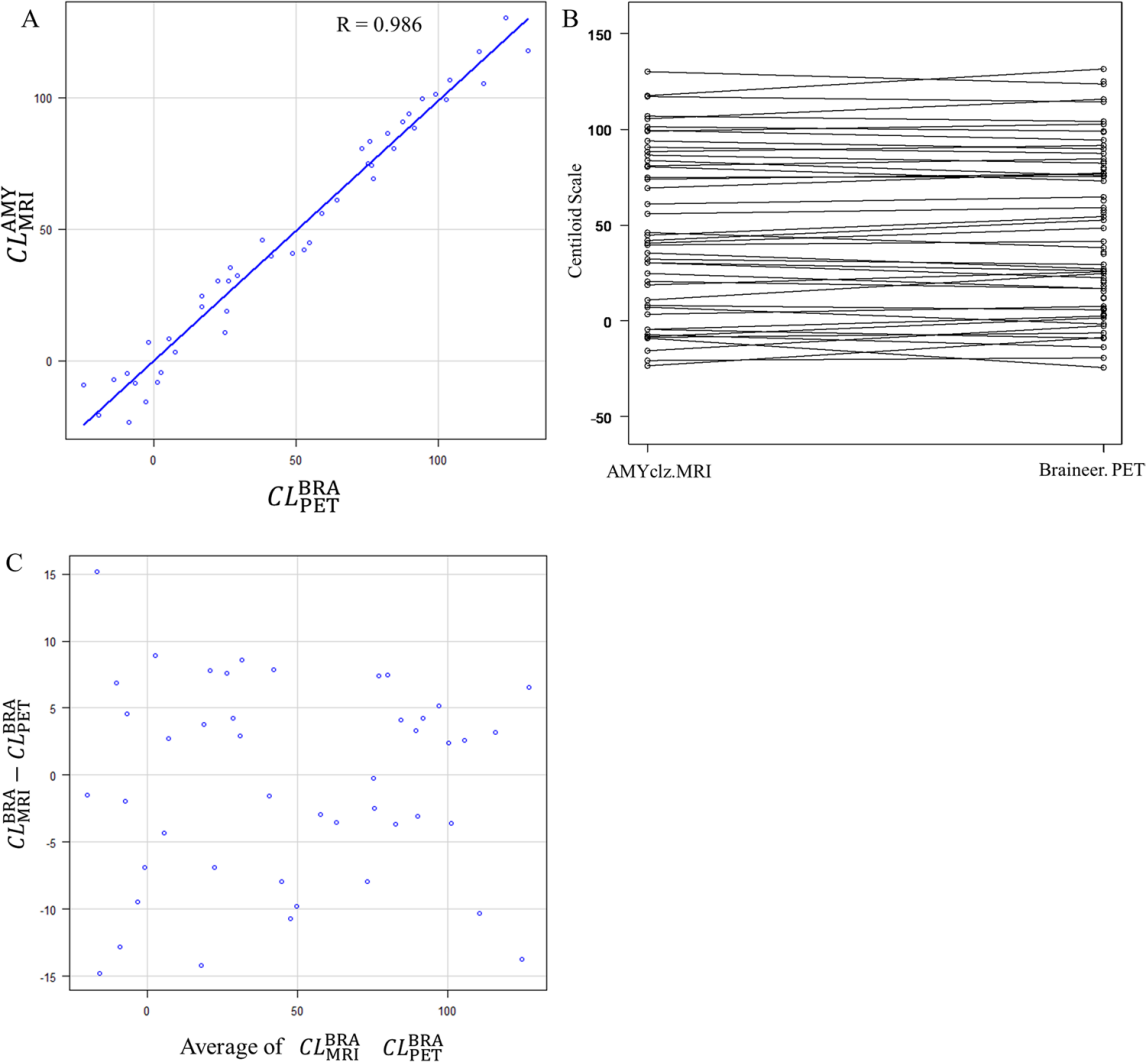
Comparison between BRAINEER^®^ Model A analysis with MRI and BRAINEER^®^ Model A analysis with PET-only. A strong correlation was observed, with a correlation coefficient of 0.970 (**A**) the Bland**–**Altman plots also revealed substantial chance errors. However, there was no discernible systematic errors (**B** and **C**)

- CT scan vs. MRI (*r* = 0.994) (Fig. 2).

- By BRAINEER^®^ Model A with MRI vs. PET-only (*r* = 0.970) (Fig. 3).

The Bland–Altman plot showed a tendency toward underestimation with CT scans when using AMYclz^®^ Neuro (mean on MRI: 48.1 ± 43.7 CL, mean on CT: 40.2 ± 41.7 CL, P-value = 8.95e-11, mean difference − 5.9 ± 4.8 CL). Furthermore, when using BRAINEER^®^ Model A, the difference between MRI and PET-only was not significant (MRI mean: 51.4 ± 45.2 CL, PET-only mean: 48.2 ± 42.1 CL, P-value = 0.192, mean difference: 2.10 ± 11.0 CL).

**Fig. 4:**
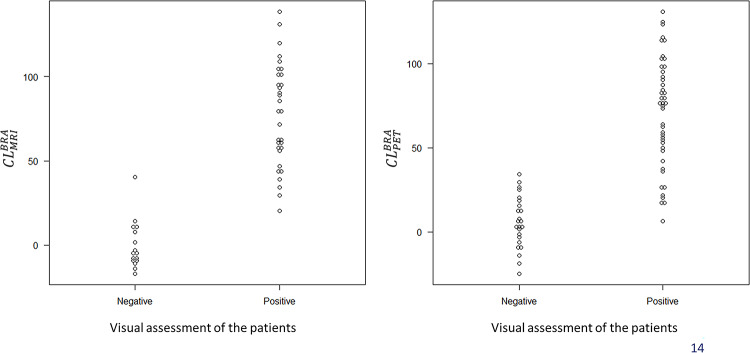
Stratifying based on visual assessment positivity/negativity and comparing CL between MRI and PET-only. Based on visual evaluation results, PET-only showed an overlap between positive and negative findings compared to the use of MRI

The CL scale had a consistent underestimation when using CT scan compared with MRI. When stratifying based on visual assessment positivity/negativity and comparing CL between MRI and PET-only, the CL calculated using PET-only showed overlap with visual assessment positivity/negativity (Fig. 4).

This result demonstrates that MRI-based standardization offers superior separation between visually positive and negative cases, whereas PET-only standardization yields overlapping CL values, particularly in borderline ranges.

Omitting MRI-based anatomical standardization was difficult in borderline cases. On the other hand, the use of commercial software produced results in accordance with previous reports. Anatomical standardization may be omitted in cases with clearly positive or negative findings.

## Discussion

**Fig. 5:**
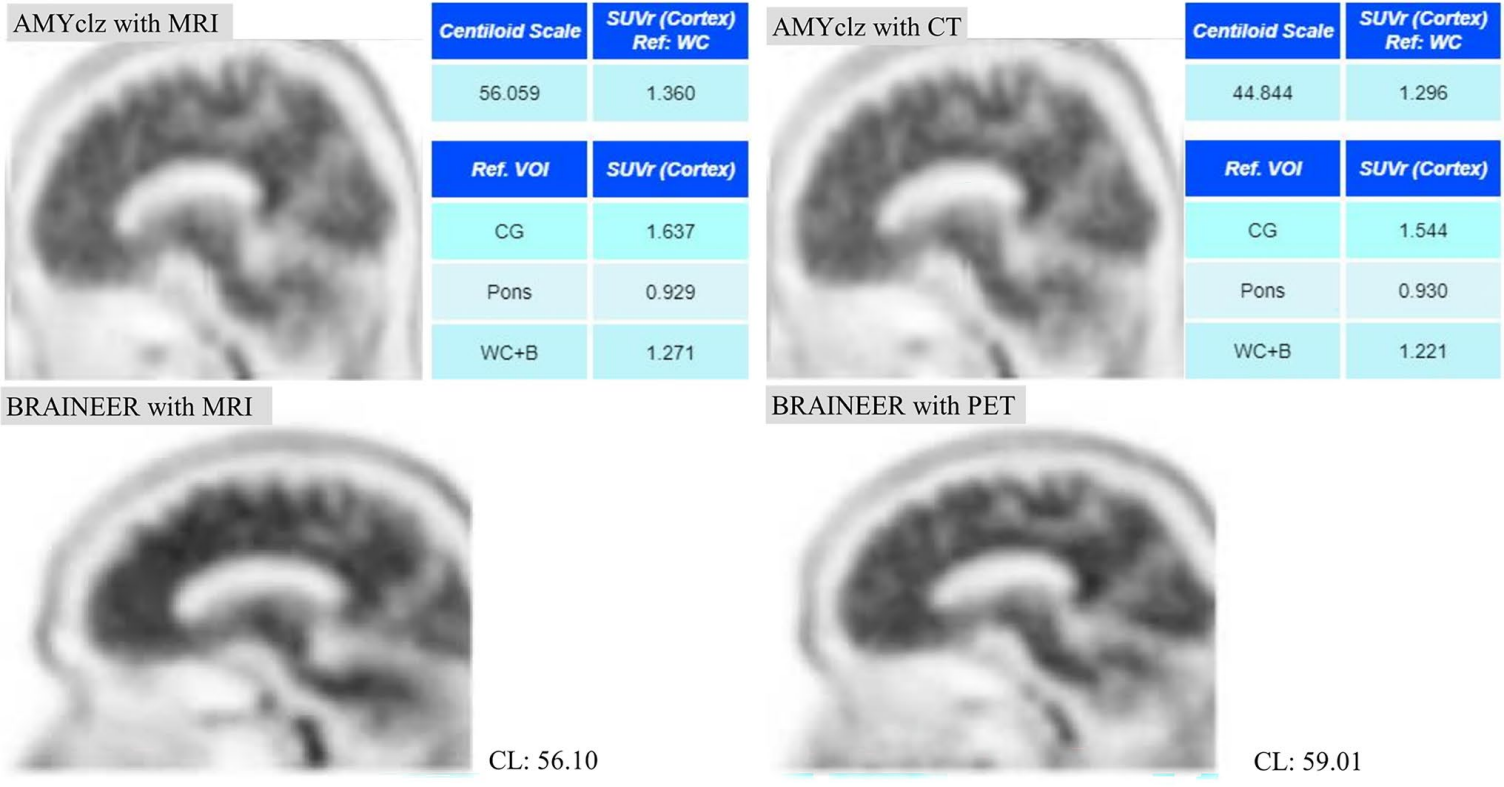
Analysis results by software for the same case, using anatomically standardized PET images and AMYclz^®^ Neuro. Following anatomical standardization, CT-based imaging showed slightly higher cerebellar uptake than MRI-based imaging in this case. Considering the unchanged SUVr in the pons, it was presumed that cerebellar uptake was elevated on CT-based imaging

This study evaluated the impact of anatomical standardization using CT scan, MRI, and PET-only methods for calculating the CL scale in amyloid PET imaging with [^18^F]florbetapir, utilizing a commercially available software. The findings showed that the CL values calculated with the CT images were more likely to be slightly underestimated compared with MRI- approaches, which is consistent with previous studies [8]. One potential reason for this underestimation is a difference in the cerebellar uptake, which can be the reference region for the CL calculation. If CT scan is used for anatomical standardization, the standardized PET images were more likely to show slightly higher uptake values in the cerebellum compared with MRI-based standardization, thereby leading to a slight underestimation of the overall CL value [8, 15]. In this study as well, some cases showed increased cerebellar uptake on CT, which may have led to underestimation of CL. (Fig. 5)

**Fig. 6:**
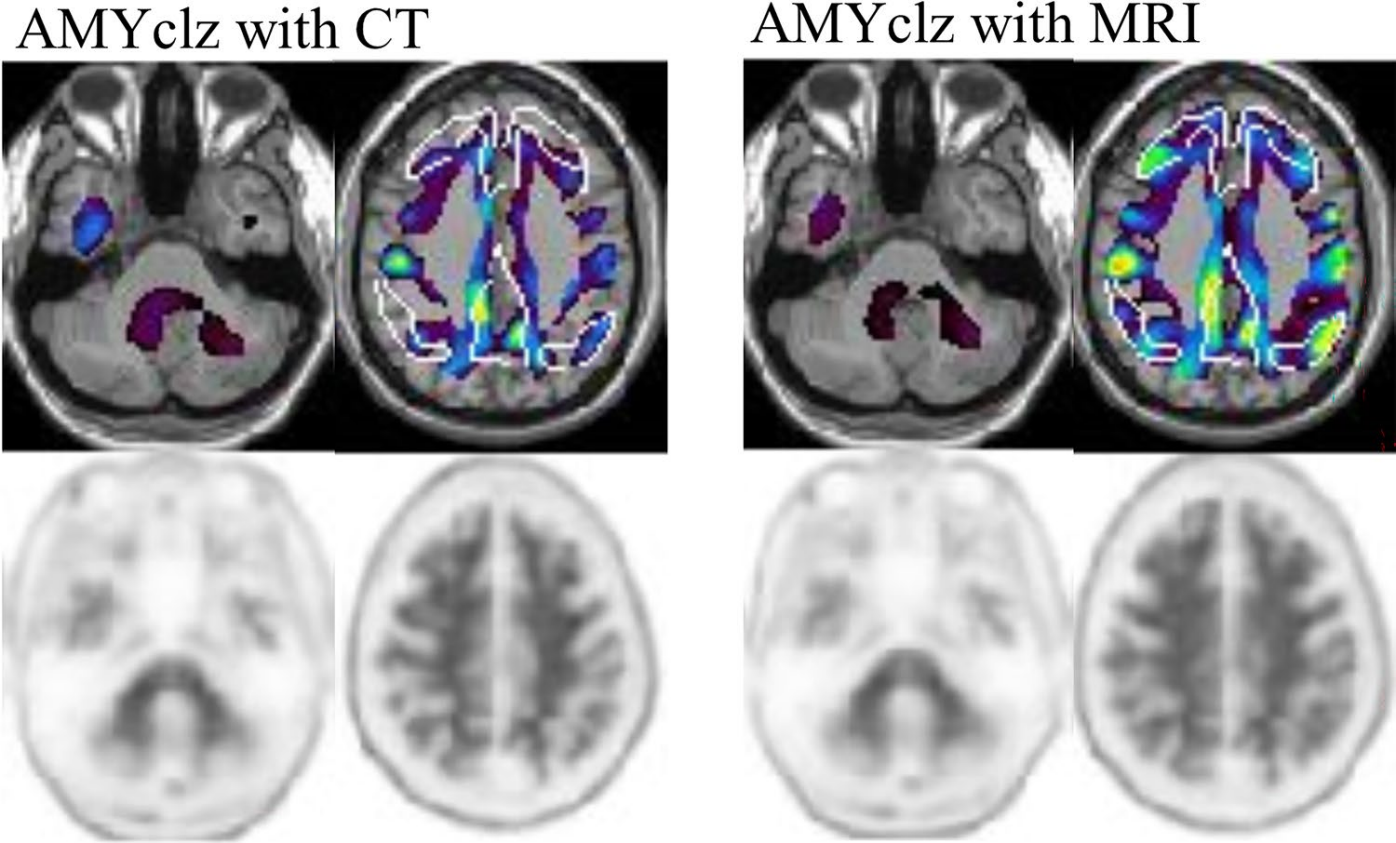
A case where cerebral cortex accumulation appeared to change due to anatomical standardization techniques. The increased accumulation in the precuneus, which was unclear on CT-based imaging, was clearly visible on MRI-based imaging

Additionally, in other cases, anatomical normalization on MRI clarified cortical uptake in the precuneus, suggesting MRI’s superior cortical-white matter resolution may have influenced the findings. (Fig. 6)

A mean difference of − 5.9 ± 4.8 CL units when using CT scan compared with MRI emphasizes the importance of anatomical reference selection, particularly in borderline cases. Nevertheless, in cases with clearly positive (CL ≥ 50) or clearly negative (CL ≤ 5) values, CL calculation based on the PET-only approach provided sufficient clinical reliability, indicating that anatomical standardization using CT scan or MRI may not be essential in such situations. According to Takenaka et al., who compared the visual evaluation consistency of amyloid PET among readers, images that were inconsistent among readers had a moderate CL [20]. As in visual evaluation, this indicates that PET-only approaches may be sufficient for specific clinical applications, particularly when a commercial software provides robust results with minimal error.

Strong correlations were observed between the different methodologies (CT scan vs. MRI: *r* = 0.994, and BRAINEER^®^ Model A with MRI vs. PET-only: *r* = 0.970), thereby supporting the reliability of the CT-based and PET-only CL calculations for clinical use. On the other hand, in our study, when using visual assessment-based positive/negative criteria, we observed overlap in CL between visual positives and negatives when calculating CL using PET-only compared to when using MRI. The aforementioned study by Takenaka et al. also demonstrated the difficulty of assessment in cases with moderate CL, which is similar to the findings of this study [20]. For borderline cases where precise quantification is important—such as in early diagnosis or longitudinal follow-up—MRI-based standardization is still preferred.

The current study had several limitations. First, the proprietary nature of the commercial software prevented full disclosure of the processing pipelines, making it difficult to verify the sources of the discrepancies. For example, the PET templates used in the BRAINEER^®^ Model A have not been publicly disclosed. Second, the MRI data were heterogeneous, with variations in the scanner models, imaging protocols, and acquisition times. Third, the relatively small number of borderline cases limited our ability to comprehensively assess the need for morphological images for these cases. Further, the retrospective design might have introduced selection bias, and variations in imaging quality could have influenced the results.

CT-based and PET-only CL calculations can be practical alternatives when contemporaneous 3D-T1WI is not available. However, users should be aware that CT scan is more likely to slightly underestimate and PET-only approaches tend to slightly underestimate CL values relative to MRI. This consideration is important when using CL values for quantitative treatment monitoring. Our study did not include pre- and posttreatment comparisons within the same patients. Thus, the application of CT scan-based or PET-only CL calculations for treatment evaluation should be further prospectively investigated.

## Conclusion

The CL value was more likely to be slightly underestimated when calculated using CT scan-based standardization and slightly underestimated when calculated using PET-only standardization, compared with MRI-based standardization. Previous studies using [^18^F]flutemetamol have reported similar findings. However, the current study specifically showed that such tendencies were also applicable to [^18^F]florbetapir PET imaging. In cases with evidently positive or negative findings, the PET-only approach can have a clinically acceptable reliability, thereby potentially streamlining the workflow when contemporaneous 3D T1WI is not available. Nevertheless, when a precise quantification is required—particularly for treatment monitoring—cautious attention must be given to the anatomical standardization method. CT-based and PET-only methods can be practical alternatives for [^18^F]florbetapir PET, provided that their systematic biases are properly considered.

## Supplementary Information

Below is the link to the electronic supplementary material.

Supplementary Material 1

## Data Availability

The datasets generated and/or analyzed during the current study are available from the corresponding author upon reasonable request.

## References

[1] Rajmohan R, Reddy PH. Amyloid-Beta and phosphorylated Tau accumulations cause abnormalities at synapses of alzheimer’s disease neurons. J Alzheimers Dis. 2017;57(4):975–99. 27567878 10.3233/JAD-160612 PMC5793225

[2] Mattsson N, et al. Staging β-Amyloid pathology with amyloid positron emission tomography. JAMA Neurol. 2019;76(11):1319–29. 31314895 10.1001/jamaneurol.2019.2214 PMC6646987

[3] Smith AM, et al. The RSNA QIBA profile for amyloid PET as an imaging biomarker for cerebral amyloid quantification. J Nucl Med. 2023;64(2):294–303. 36137760 10.2967/jnumed.122.264031 PMC9902844

[4] Klunk WE, et al. The centiloid project: standardizing quantitative amyloid plaque Estimation by PET. Alzheimers Dement. 2015;11(1):1–e151. 25443857 10.1016/j.jalz.2014.07.003 PMC4300247

[5] Collij LE, et al. Centiloid recommendations for clinical context-of-use from the AMYPAD consortium. Alzheimer’s Dement. 2024;20(12):9037–48. 39564918 10.1002/alz.14336 PMC11667534

[6] Yokoyama K, et al. Computed-tomography-guided anatomic standardization for quantitative assessment of dopamine transporter SPECT. Eur J Nucl Med Mol Imaging. 2017;44(3):366–72. 27544223 10.1007/s00259-016-3496-0

[7] Alae Eddine EB, et al. CT-guided spatial normalization of nuclear hybrid imaging adapted to enlarged ventricles: impact on striatal uptake quantification. Neuroimage. 2024;294:120631. 38701993 10.1016/j.neuroimage.2024.120631

[8] Matsuda H, et al. Amyloid PET quantification using low-dose CT-guided anatomic standardization. EJNMMI Res. 2021;11(1):125. 34905145 10.1186/s13550-021-00867-7 PMC8671596

[9] Imabayashi E, et al. Comparison between brain CT and MRI for voxel-based morphometry of alzheimer’s disease. Brain Behav. 2013;3(4):487–93. 24381817 10.1002/brb3.146 PMC3869687

[10] Bourgeat P, et al. Implementing the centiloid transformation for 11 C-PiB and β-amyloid 18F-PET tracers using CapAIBL. Neuroimage. 2018;183:387–93. 30130643 10.1016/j.neuroimage.2018.08.044

[11] Shimokawa N, et al. Feasibility study of a PET-only amyloid quantification method: a comparison with visual interpretation. Ann Nucl Med. 2020;34(9):629–35. 32535743 10.1007/s12149-020-01486-3

[12] Imabayashi E, et al. Automated semi-quantitative amyloid PET analysis technique without MR images for Alzheimer’s disease. Ann Nucl Med. 2022;36(10):865–75. 35821311 10.1007/s12149-022-01769-x PMC9515054

[13] Edison P, et al. Comparison of MRI based and PET template based approaches in the quantitative analysis of amyloid imaging with PIB-PET. Neuroimage. 2013;70:423–33. 23261639 10.1016/j.neuroimage.2012.12.014

[14] Shang C, et al. Comparison of consistency in centiloid scale among different analytical methods in amyloid PET: the CapAIBL, VIZCalc, and amyquant methods. Ann Nucl Med. 2024;38(6):460–7. 38512444 10.1007/s12149-024-01919-3 PMC11108942

[15] Matsuda H, et al. Development of software for measuring brain amyloid accumulation using 18F-florbetapir PET and calculating global centiloid scale and regional Z-score values. Brain Behav. 2023;13(7):e3092. 37287410 10.1002/brb3.3092 PMC10338776

[16] Ashburner J, Friston KJ. Nonlinear spatial normalization using basis functions. Hum Brain Mapp. 1999;7(4):254–66. 10408769 10.1002/(SICI)1097-0193(1999)7:4<254::AID-HBM4>3.0.CO;2-G PMC6873340

[17] Ashburner J. A fast diffeomorphic image registration algorithm. Neuroimage. 2007;38(1):95–113. 17761438 10.1016/j.neuroimage.2007.07.007

[18] Collignon A, et al. Automated multi-modality image registration based on information theory. Kluwer Academic. 1995.

[19] Navitsky M, et al. Standardization of amyloid quantitation with Florbetapir standardized uptake value ratios to the centiloid scale. Alzheimer’s Dement. 2018;14(12):1565–71. 30006100 10.1016/j.jalz.2018.06.1353

[20] Takenaka A, et al. Interrater agreement and variability in visual reading of [18F] flutemetamol PET images. Ann Nucl Med. 2025;39(1):68–76. 39316332 10.1007/s12149-024-01977-7 PMC11706841

