# Supplementary material for: Comparison of MRI-, CT- and PET-based anatomical standardization for Centiloid scale calculation in [18F]florbetapir positron emission tomography
Source: Ann Nucl Med. 2025 Dec 4;40(3):349–56. doi: 10.1007/s12149-025-02134-4 (PMC12935794; doi:10.1007/s12149-025-02134-4)
Supplement: Supplementary file 1 — Supplementary Material 1 [file 12149_2025_2134_MOESM1_ESM.docx]

Supplement Table 1: MRI scanners, principal imaging parameters, and number of cases examined on each system in this study

| **MRI Model** | **Manufacturer (City, Country)** | **No. of Participants** | **Field Strength** | **Flip Angle (°)** | **TR (msec)** | **TE (msec)** | **FOV (cm)** |
| --- | --- | --- | --- | --- | --- | --- | --- |
| Discovery MR750w | GE Healthcare (Chicago, Illinois, USA) | 29 | 3T | 14 | 6.7 | 2.6 | 26 |
| MAGNETOM Spectra | Siemens Healthineers (Erlangen, Germany) | 5 | 3T | 9 | 1800 | 2.8 | 24 |
| Signa HDxt | GE Healthcare (Chicago, Illinois, USA) | 4 | 3T | 90 | 600 | 10.3 | 26 |
| MAGNETOM Sola | Siemens Healthineers (Erlangen, Germany) | 2 | 1.5T | 120 | 500 | 26 | 24 |
| Vantage Orian | Canon Medical Systems Corporation (Otawara, Tochigi, Japan) | 2 | 1.5T | 20 | 15 | 5.5 | 23 |
| TRILLIUM Oval | Fujifilm Healthcare Corporation (Tokyo, Japan) | 2 | 3T | 90 | 450 | 6.4 | 26 |
| MAGNETOM Skyra | Siemens Healthineers (Erlangen, Germany) | 1 | 3T | 8 | 2300 | 2.3 | 26 |
| SIGNA Voyager | GE Healthcare (Chicago, Illinois, USA) | 1 | 1.5T | 25 | 12.3 | 5.1 | 24 |
| Brivo MR335 | GE Healthcare (Chicago, Illinois, USA) | 1 | 1.5T | 25 | 12.7 | 5.2 | 24 |
